# The effect of processing partial information in dynamic face perception

**DOI:** 10.1038/s41598-024-58605-7

**Published:** 2024-04-29

**Authors:** Nihan Alp, Gülce Lale, Ceren Saglam, Bilge Sayim

**Affiliations:** 1 Psychology, Sabanci University, Istanbul, Türkiye; 2 Graduate School of Systemic Neurosciences, Ludwig-Maximilians-Universität München, Munich, Germany; 3 Department of General Psychology, University of Padua, Padova, Italy; 4 Univ. Lille, CNRS, UMR 9193, SCALab – Sciences Cognitives et Sciences Affectives, F – 59000, Lille, France

**Keywords:** Dynamic face perception, Holistic representation, Temporal integration, Psychology, Perception

## Abstract

Face perception is a major topic in vision research. Most previous research has concentrated on (holistic) spatial representations of faces, often with static faces as stimuli. However, faces are highly dynamic stimuli containing important temporal information. How sensitive humans are regarding temporal information in dynamic faces is not well understood. Studies investigating temporal information in dynamic faces usually focus on the processing of emotional expressions. However, faces also contain relevant temporal information without any strong emotional expression. To investigate cues that modulate human sensitivity to temporal order, we utilized muted dynamic neutral face videos in two experiments. We varied the orientation of the faces (upright and inverted) and the presence/absence of eye blinks as partial dynamic cues. Participants viewed short, muted, monochromic videos of models vocalizing a widely known text (National Anthem). Videos were played either forward (in the correct temporal order) or backward. Participants were asked to determine the direction of the temporal order for each video, and (at the end of the experiment) whether they had understood the speech. We found that face orientation, and the presence/absence of an eye blink affected sensitivity, criterion (bias) and reaction time: Overall, sensitivity was higher for upright compared to inverted faces, and in the condition where an eye blink was present compared to the condition without an eye blink. Reaction times were mostly faster in the conditions with higher sensitivity. A bias to report inverted faces as ‘backward’ observed in Experiment I, where upright and inverted faces were presented randomly interleaved within each block, was absent when presenting upright and inverted faces in different blocks in Experiment II. Language comprehension results revealed that there was higher sensitivity when understanding the speech compared to not understanding the speech in both experiments. Taken together, our results showed higher sensitivity with upright compared to inverted faces, suggesting that the perception of dynamic, task-relevant information was superior with the canonical orientation of the faces. Furthermore, partial information coming from eye blinks, in addition to mouth movements, seemed to play a significant role in dynamic face perception, both when faces were presented upright and inverted. We suggest that studying the perception of facial dynamics beyond emotional expressions will help us to better understand the mechanisms underlying the temporal integration of facial information from different -partial and holistic- sources, and that our results show how different strategies, depending on the available information, are employed by human observers when judging the temporal order of faces.

## Introduction

Human faces contain a wealth of information, ranging from sex and age to ethnicity, emotional states, and identity^1–7^. The process of face perception helps us to recognize subtle emotional expressions and facilitates speech comprehension, enhancing communication^8,9^. Failures to process faces can lead to serious consequences, such as not being able to recognize one’s relatives or failing to understand nonverbal cues during face-to-face communication^10,11^.

One well-defined property of face processing is holistic face perception^12^. Previous research has demonstrated that faces are processed holistically: the human brain processes faces as an integrated whole rather than separate individual features such as eyes, nose, and mouth^7,13^. Specifically, holistic face processing refers to the (automatic) integration of part-based information. To investigate holistic face processing, the composite face paradigm has been widely used^14,15^. In this paradigm, participants are presented with a face composed of the top and bottom parts of either the same face (congruent) or two different faces (incongruent) creating a new composite face. Participants are instructed to focus on either the top or bottom half of the composite face and disregard the unattended part. When asked to identify the face, participant perform better when the two halves belong to the same compared to different individuals. These studies demonstrate that the irrelevant part of the face -despite instructions not to attend to it- still plays a role in modulating face perception^7,14,15^. Interestingly, the holistic processing of faces is significantly diminished when faces are inverted (i.e., presented upside-down). One suggestion for the reduction of the face composite effect with inverted faces is that the unnatural view shifts attention to part-based cues as present in the eyes and mouth^16^.

While several studies have investigated face processing by focusing on the face composite effect, they have mainly used static stimuli such as photographs or cartoons of different faces. The prominent use of static stimuli was due to, for example, technological limitations in the past, and the suitability of static stimuli for detecting brain areas sensitive to processing faces^3^. However, compared to dynamic faces, static faces lack many characteristics of faces occurring in real life, where faces are characterized by various expressions and their transitions, lip and eye movements, as well as head movements. Previous studies have also revealed that different brain regions are activated when processing dynamic (e.g., superior temporal sulcus, STS^17^) and static (e.g., fusiform face area, FFA^18^) face stimuli^3,19,20^.

The use of dynamic face stimuli not only increases the ecological validity of results but also enables researchers to address questions that cannot be answered by using static face images^3,21,22^. For example, Reinl and Bartels (2015) investigated to what extent observers can discriminate the temporal sequence of facial movement^23^. They asked actors to display increasing and decreasing fear using facial expressions. Observers were shown video recordings of these facial movements in either a natural (forward) or an artificial (backward) timeline and asked to rate the emotional intensities, artificialness, and convincingness of the expression^23^. The results showed that participants rated the artificialness of videos in backward conditions significantly higher and the convincingness significantly lower compared to videos in forward conditions. Another study, conducted by the same researchers found that the FFA was predominantly active when processing emotional facial expressions in the natural timeline, while the STS was predominantly active when processing an artificial timeline; however, only for decreasing emotional strength^8^. Cunningham and Wallraven (2009) used dynamic face stimuli to demonstrate that people’s recognition of emotional expressions (e.g., sad, happy, etc.) of dynamic faces shown in a natural timeline was significantly higher than the recognition rate of dynamic faces shown in an artificial timeline^24^. Furthermore, Alp and Ozkan (2022) investigated the underlying neural correlates of temporal integration processes during dynamic face perception using dynamic face videos. Unlike previous studies that focused on emotional facial expressions, they used dynamic face videos that featured models vocalizing a text with a neutral facial expression^25^. Participants were presented with models’ dynamic face videos that were either played forward or backward in time. Even and odd frames of the dynamic face videos were presented with different contrast levels at distinct frequencies (e.g. even frames at $f_1$ and odd frames at $f_2$ Hz) to investigate temporal integration processes during dynamic face perception. By tagging even and odd frames with two frequencies, it was possible to pinpoint temporal integration processes at distinct frequencies (i.e. intermodulation (IM) frequencies = $mf_1\pm nf_2$) in the electroencephalography (EEG) signals. Identifying discernible IMs in the frequency spectrum alone can serve as an indicator that the time between frames (1 / 60 = 0.016 seconds) is adequate for detecting the underlying neural correlates of temporal integration. Importantly, there was a clear dissociation between forward and backward face videos in the left occipital and medial frontal regions which are involved in processing dynamic faces^26,27^.

While there is an increasing body of research on dynamic face perception, most studies have explored dynamicity through variations in emotional facial expressions^8,23^. Hence, these studies cannot distinguish dynamic features of face processing from the processing of emotional expressions. Furthermore, various studies investigating temporal integration in face perception have focused on determining whether presenting face parts (i.e. mouth and eyes) separately can shed light on the question of whether temporal integration reflects holistic processing^28,29^. This line of research is probing whether observing distinct face parts sequentially still leads to an integrated perception of the face, emphasizing the importance of temporal dynamics in understanding holistic processing in face perception. These studies focus on how temporally separated face parts influence holistic processing, a key aspect of configural face perception. Moreover, it has been demonstrated that part-based information is not only beneficial but equally important as configural information in enhancing face processing^16,30,31^. Dedicated neural populations are responsible for encoding information from salient facial features such as the eyes and mouth^32,33^. However, the processing of temporal order when perceiving dynamic faces remains elusive.

In particular, how the visual system integrates information from different -partial and holistic- sources in dynamic face perception is unknown. In this study, we define holistic representation of temporal order as follows: it refers to the perceptual integration of temporally separated parts into a normal chronological sequence, enabling a comprehensive understanding of the entire temporal structure and the relationships between the constituent components within that temporal order. Understanding this process is essential for elucidating how our brains organize and interpret the progression of events, aiding efficient information processing and decision-making based on temporal contexts. We hypothesize that face orientation may influence the sensitivity, bias and reaction time of discriminating the temporal order of dynamic faces (forward or backward), and that the part-based information from mouth movements and eye blinks may provide essential information about the temporal order that can be flexibly used by human observers^34^. In this study, we use neutral facial expressions (of models reciting a text) with the aim of excluding highly salient facial muscle movements involved in expressing emotions. When allowing for emotional expressions, judgments of the temporal order would exclusively (or at least to a large extent) be based on these expressions and not on subtle mouth movements or eye blinks as targeted in the current study. Hence, our objective is to use a set of stimuli where emotional expressions do not dominate participants’ responses. To uphold ecological validity, our manipulation exclusively focused on the presence or absence of eye blink information in the presented dynamic neutral faces. We hypothesize that orientation influences the sensitivity and reaction time of detecting the temporal order, and the presence/absence of an eye blink provides essential information about the temporal order, thereby affecting participants’ sensitivity and reaction time. To test these hypotheses, we utilized 3-second video clips from The Sabancı University Dynamic Face (SUDFace) Database^35^. We created a $2 \times 2$ factorial design, manipulating the Orientation (“Upright” vs “Inverted”), and the Presence/Absence of an Eye Blink (“With Blink” vs “No Blink”). Participants were presented with short, muted, monochromatic videos and asked to indicate whether the videos were presented in a forward or backward temporal order. We analyzed participants’ sensitivity ($d^\prime$), bias (C), and reaction time (RT) to explore sensitivity to temporal order (“forward” vs “backward”) when processing dynamic faces and the influence of partial dynamic cues on this process. Our results suggest that participants flexibly use partial information from different sources to maximize performance. Importantly, we found that the face-inversion effect also holds for dynamic, predominantly partial cues, suggesting that factors such as familiarity and processing efficiency yield relative facilitation when viewing faces in their canonical form compared to inverted orientation.

## Methods

**Figure 1:**
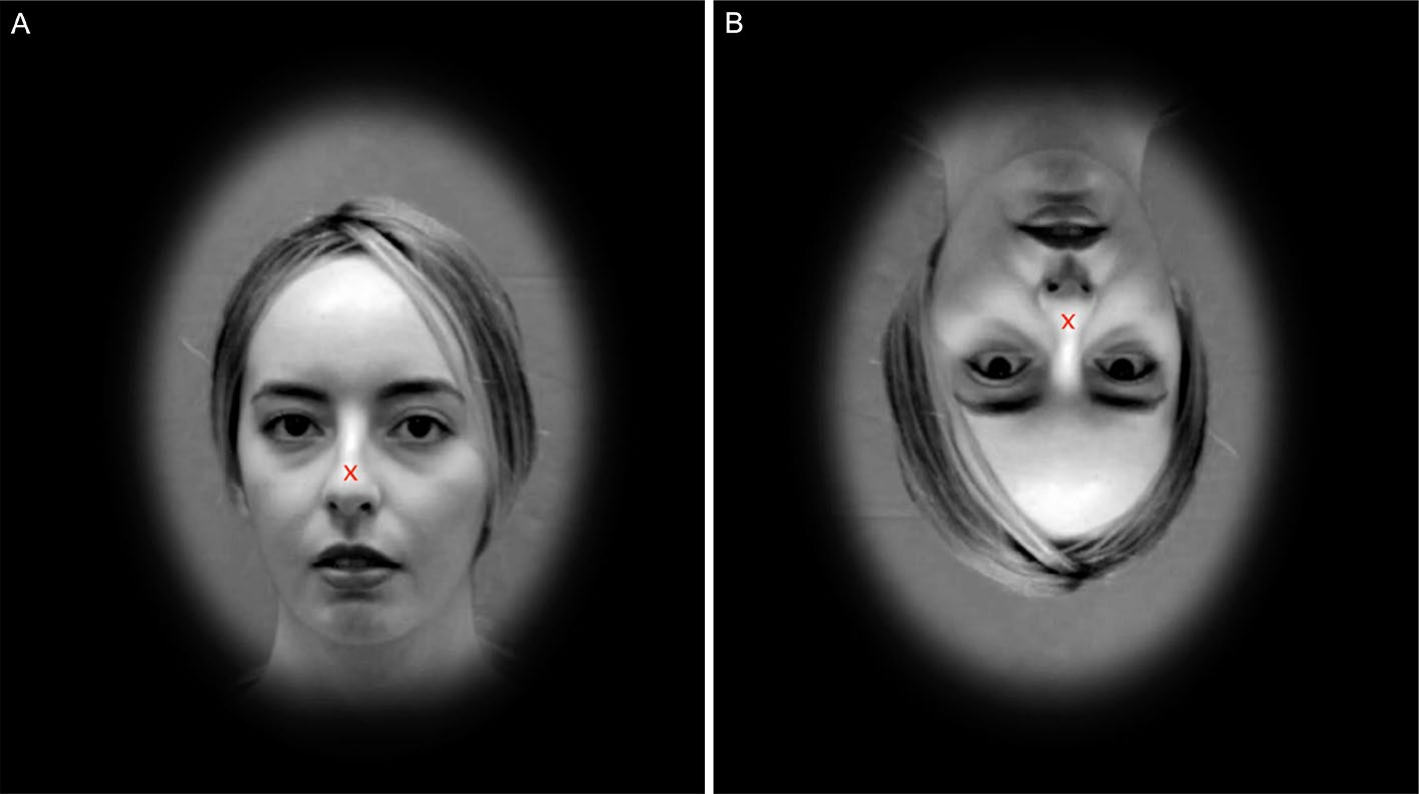
Examples of upright (**A**) and inverted (**B**) faces that were used in the experiment. The fixation cross was located equidistant from the eyes and mouth regions of the stimuli. (Faces can be shown here as the models agreed with the publication of their images before the experiment).

The stimuli consisted of short muted monochromic dynamic face videos, each lasting 3 seconds, in which models vocalized the Turkish National Anthem, with a neutral face. The videos were taken from the SUDFace Database^35^.To ensure a balanced sex ratio, we included 14 videos of females and 14 videos of males in the main experiment. During the training session, we used one female and one male video, both of which were not included in the main experiment. These videos were also taken from the SUDFace Database^35^. Prior to the experiment, informed consent was obtained from all models for the publication of individual information/images. We created an elliptical mask that cropped the outline of the faces depending on the models’ face length and the coordination of their noses to place the head in the middle of the ellipse such that the cropped video contained only the head and the neck. We also converted the videos to grayscale using the SHINE toolbox^36^ to equate low-level properties (see Fig. 1).

To manipulate partial dynamic cues (with blink or no blink), we used clips from the SUDFace Database^35^ videos in which models did not blink for 3 seconds. To generate the stimulI with an eye blink, we used clips in which the models blinked once within 3 seconds. To facilitate participants’ attention to both, the eyes and mouth regions, we placed a fixation cross approximately equidistant from the mouth and the nose, as shown in Fig. 1. The distance between the eyes and mouth was computed by two research assistants utilizing SUDFace Database^35^ videos. All the videos had a frame rate of 60 Hz (for details see the following section: Experimental procedure).

### Experimental procedure

We conducted two experiments (Experiment I and II) to examine the effects of two factors (Orientation, Presence/Absence of an Eye Blink) on dynamic face perception. Since models in the SUDFace Database^35^ included Sabancı University students, to avoid participants recognizing the models, both experiments were conducted online using Labvanced^37^.

Each experiment was divided into three phases: a training session with 16 training videos, the main experiment with 208 videos in total, and questionnaires with three questions. Before starting the experiment, participants were instructed to use a credit card method^37^ to adjust stimulus size. Participants completed an eye-tracking calibration to ensure that they kept their eyes on the fixation point during the experiment. Participants were asked to sit at an arm’s distance (approximately 57 cm) throughout the experiment. Additionally, participation in the experiment required the use of a laptop or desktop computer with a standard monitor. Access via tablet or phone was not permitted. Using these setups and parameters, we presented stimuli at a size of approximately $17^{\circ }$ $\times$ $11^{\circ }$ of visual angle for each participant. Eye movements were recorded during the experiment with a webcam-based eye tracking algorithm method which yields accurate results also when experiments are conducted remotely^38^. In the training session (16 trials), participants were presented with 3-second dynamic face videos. The task was to indicate whether the video was played forward or backward, using the right and left arrow keys on the keyboard (right to indicate forward, and left to indicate backward). Following each training video, the correct response was displayed for 1500 ms to provide feedback to participants. The training videos were not included in the main experiment.

In the main experiment, participants performed the same task as in the training session, indicating whether the presented stimulus was played in normal chronological order (forward) or reversed order (backward). There was no feedback. There were four conditions, in which we manipulated the Orientation of the faces (“Upright” vs. “Inverted”), and the part-based information coming from eyes: Presence/Absence of an Eye Blink (“With Blink” vs. “No Blink”). Participants were instructed to respond after the stimulus offset to ensure equal processing time for all participants.

At the end of each experiment, all participants were required to complete a questionnaire consisting of three questions (see Supplementary Questionnaire). The first two questions aimed to elicit insight into how participants determined whether a video was played forward or backward. The third question assessed participants’ comprehension of the speech presented in the video. Participants who understood the speech were also asked about the content of the speech.

## Experiment I: random order

**Figure 2:**
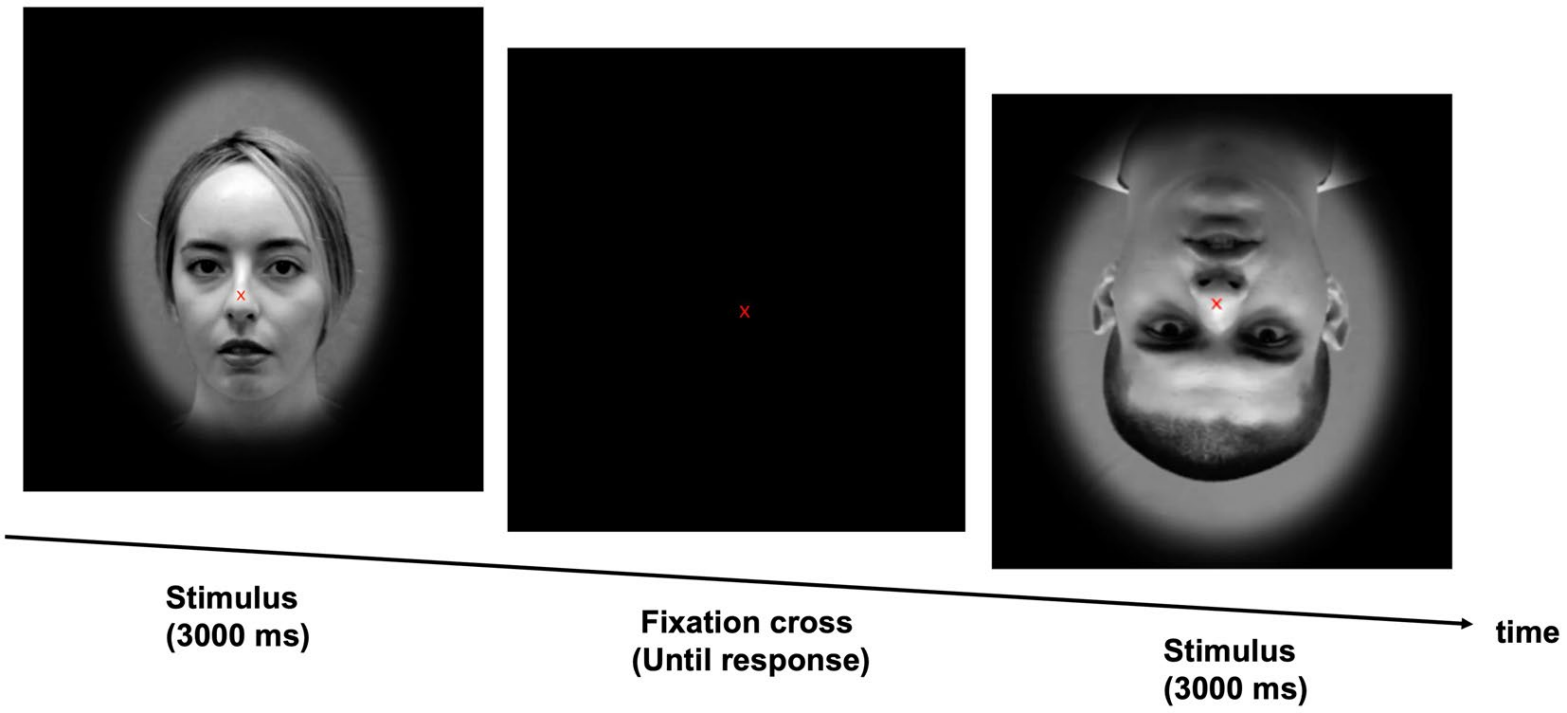
Illustration of the experimental procedure. Each video was presented for 3 s, followed by a blank screen with fixation cross until the participant’s response. The next stimulus was presented immediately after the participant’s response (approximately 10 ms system delay).

In Experiment I, the two factors Presence/Absence of an Eye Blink and Orientation were randomized. In total, each participant completed 224 trials (16 trials in the training session and 208 trials in the main experiment; i.e., 26 trials per condition) in a single run. During the training session and main experiment, participants were asked to fixate their eyes on the fixation cross presented equally distant to the eye and mouth region of the dynamic face videos (see Fig. 1). After each stimulus, the fixation cross remained on a black screen. Overall, the experiment lasted approximately 30 minutes, encompassing the training session, the main experiment and the questionnaire. An illustration of the experiment is provided in Fig. 2.

### Participants

A total of 62 students with normal or corrected-to-normal vision (39 females, 23 males, mean age: 22.6) participated in the study in exchange for research credits. Nine participants were excluded from the original 62 due to failure to follow the instructions correctly, as revealed by the answers to the questionnaire at the end of the experiment. Of the remaining 53 participants, one was an Urdu speaker, two of them were English speakers and the others were native Turkish speakers. The three participants who were not native Turkish speakers were excluded to eliminate linguistic disparities (since individuals proficient in Turkish are usually familiar with the Turkish National Anthem). Of the remaining 50 Turkish speakers, 4 were removed due to $d^\prime < 0$ in all conditions. We included 46 participants (27 females, 19 males: M = 20.93, SD = 1.27; M = 21.58, SD = 1.43) for further statistical analyses of the data ($d^\prime$, C and RT). One participant was excluded from the eye tracking analysis due to missing eye tracking data. The study was approved by the ethics committee of Sabancı University and conducted following their guidelines. All participants signed informed consent before the experiment.

### Results

We ran repeated-measures analyses of variance (ANOVAs) on the dependent variables sensitivity ($d^\prime$; and criterion), and reaction time (RT). All post hoc comparisons in this study were Bonferroni corrected. We utilized signal detection theory (SDT^39^) to calculate sensitivity and criterion. Sensitivity in SDT^39^ refers to the ability to accurately detect signals, and criterion (bias) refers to the tendency to favor one response over another. We defined videos played “forward” as signal (target present) and “backward” as noise (target absent). The Presence/Absence of an Eye Blink (“With Blink”, “No Blink”) and Orientation (“Upright”, “Inverted”) were within-subjects factors. Trials with reaction times faster than 100ms and slower than 3000ms (6s after stimulus onset) were excluded from the analysis in both experiments^2^. We excluded these trials to have identical viewing time and equate attention to the stimuli between trials and observers as much as possible (too fast RTs suggest early disengagement from watching the video, too slow RTs suggest not following the protocol to respond as fast and accurate as possible after stimulus offset).

### Sensitivity and criterion ($d^\prime$ and C values)

**Figure 3:**
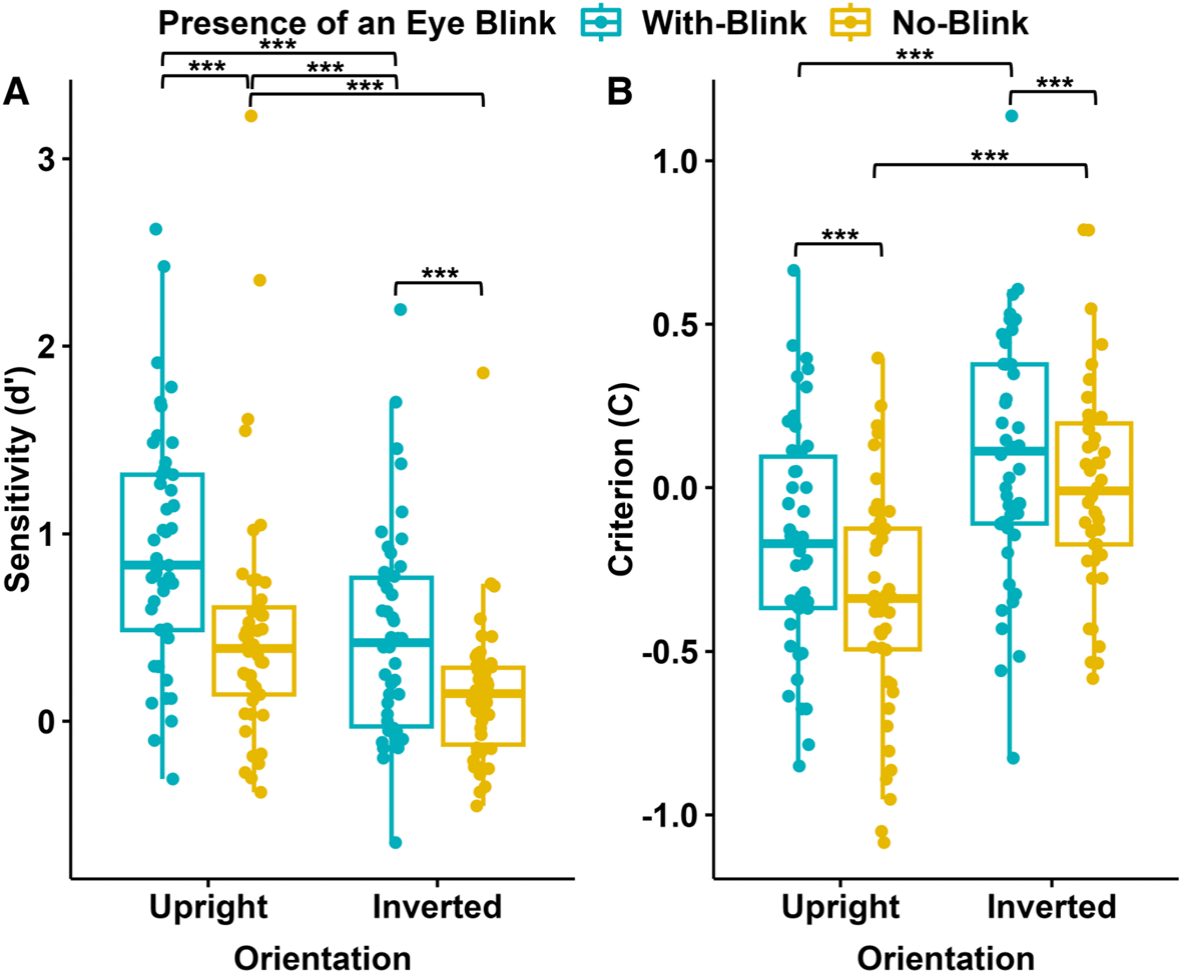
Sensitivity ($d^\prime$) and criterion (**C**) results (N = 46) of Experiment I. Blue data points represent the responses to the stimuli with eye blinks, and yellow data points represent the responses to the stimuli without eye blinks; each for upright and inverted orientations of the faces. The y-axis represents $d^\prime$ (**A**) and C values (**B**). The 75th percentile is denoted by the upper hinge, and the lower hinge corresponds to the 25th percentile. Whiskers extend to values that lie within 1.5 times the interquartile range (IQR).

A repeated-measures ANOVA was conducted to examine the effects of the Presence/Absence of an Eye Blink (“With Blink” vs “No Blink”) and Orientation (“Upright” vs “Inverted”) on participants’ sensitivity ($d^\prime$) to the Temporal Order (“forward”, “backward”). There were significant main effects of the Presence/Absence of an Eye Blink (F(1, 45) = 25.889, p $<0.001$, $\eta$^2^ = 0.158) and Orientation (F(1, 45) = 34.413, p $<0.001$, $\eta$^2^ = 0.186). There was no interaction between the Presence/Absence of an Eye Blink and Orientation (see Fig. 3). Post hoc comparisons showed that sensitivity was higher in the “With Blink” (M = 0.681, SD = 0.529) compared to “No Blink” condition (M = 0.312, SD = 0.447), and when the Orientation was “Upright” (M = 0.697, SD = 0.575) compared to “Inverted” (M = 0.296, SD = 0.369). Participants’ sensitivity was higher in the “With Blink-Upright” than in the “With Blink-Inverted” condition (Mdif = 0.463, 95% CI [0.233, 0.692], p $<0.001$) and in the “No Blink-Upright” than the “No Blink-Inverted” condition (Mdif = 0.339 95% CI [0.109, 0.596], p $<0.001$). Sensitivity was significantly different than zero in all, conditions: “With Blink-Upright” (M = 0.912, 95% CI [0.700, 1.125], p $<0.001$), “With Blink-Inverted” (M = 0.450, 95% CI [0.237, 0.662], p $<0.001$, “No Blink-Upright” (M = 0.482, 95% CI [0.269, 0.694], p $<0.001$], except for “No Blink-Inverted”(M = 0.143, 95% CI [$-0.070$, 0.355], p = 0.365).

A repeated-measures ANOVA was conducted to examine the effects of the Presence/Absence of an Eye Blink and the Orientation on participants’ C values in detecting the Temporal Order. There were significant main effects of Presence/Absence of an Eye Blink (F(1, 45) = 15.278, p $<0.001$, $\eta$^2^ = 0.054) and the Orientation (F(1, 45) = 27.994, p $<0.001$, $\eta$^2^ = 0.259). There was no significant interaction between the Presence/Absence of an Eye Blink and the Orientation. Participants’ C values were significantly higher in the “With Blink” condition (M = $-0.032$, SD = 0.287) compared to the “No Blink” condition (M = $-0.171$, SD = 0.238). Additionally, participants’ C values were significantly lower when the Orientation was “Upright” (M = $-0.254$, SD = 0.301) compared to “Inverted” (M = 0.052, SD = 0.311). Participants’ C values were lower in the “With Blink-Upright” than the “With Blink-Inverted” condition (Mdif = $-0.252$, 95% CI [$-0.428$, $-0.076$], p = 0.001) and in “No Blink-Upright” than “No Blink-Inverted” condition (Mdif = $-0.360$, 95% CI [$-0.535$, $-0.184$], p $<0.001$). The comparison of all group means to zero showed that C values were significantly lower than zero in the two "Upright" conditions: “With Blink-Upright” (M = −0.158, 95% CI [−0.286, −0.030], p = 0.009), “No Blink-Upright” (M = −0.351, 95% CI [−0.479, −0.223], p < 0.001), but not in the "Inverted" conditions: “With Blink-Inverted” (M = 0.094, 95% CI [−0.034, 0.222], p = 0.259, “No Blink-Inverted”(M = 0.009, 95% CI [−0.119, 0.137], p = 1.000).

### Reaction time (RT)

**Figure 4:**
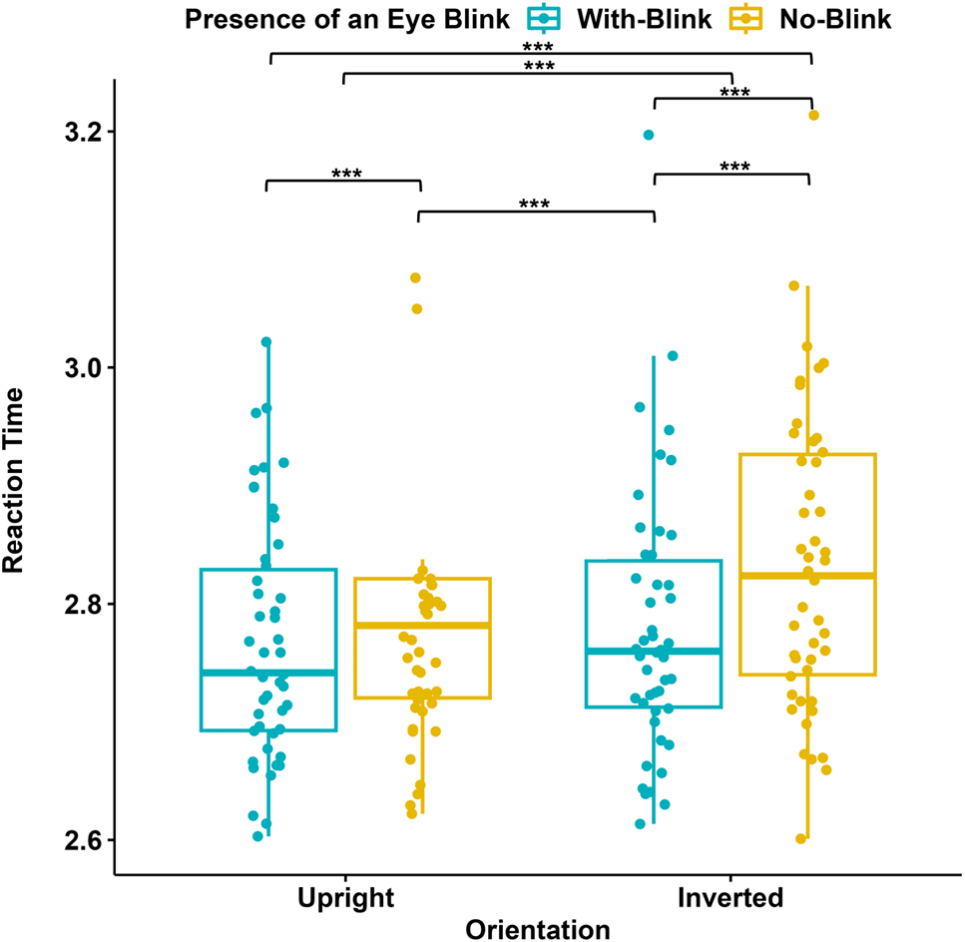
Reaction times (in seconds) in Experiment I (N = 46). Blue data points represent RTs for stimuli with eye blinks, and yellow data points represent RTs for stimuli without eye blinks; each for upright and inverted orientations of the faces. The y-axis represents RTs (log10). The 75th percentile is denoted by the upper hinge, and the lower hinge corresponds to the 25th percentile. Whiskers extend to values that lie within 1.5 times the interquartile range (IQR).

We normalized the reaction time (RT) data by applying a logarithmic transformation to normalize the skewed distribution for further statistical analyses. Next, we ran a two-way repeated measures ANOVA with the Presence/Absence of an Eye Blink and the Orientation as within-subjects factors. The results revealed that there were main effects of the Presence/Absence of an Eye Blink (F(1, 45) = 21.643, p $<0.001$, $\eta$^2^= 0.135) and the Orientation (F(1,45) = 17.902, p $<0.001$, $\eta$^2^ = 0.093) (see Fig. 4). Participants’ reaction time was faster in the “With Blink”( M = 2.773, SD = 0.102) than in the “No Blink” condition (M = 2.809, SD = 0.102) and the “Upright” (M = 2776, SD = 0.098) than in the “Inverted” condition (M = 2.806, SD = 0.112). There was no significant interaction between the Presence/Absence of an Eye Blink and the Orientation. Participants’ reaction time was faster in the “No Blink-Upright” condition than in the “No Blink-Inverted” condition (Mdif = $-0.047$, 95% CI [-0.074, $-0.020$], p $<0.001$). However, there was no significant effect of the Orientation when the blink information was present (Mdif = $-0.013$, 95% CI [$-0.040$, $-0.014$], p = 1.000).

### Language comprehension ($d^\prime$, criterion, RT)

To test whether understanding the content of the speech played a role in sensitivity, we divided participants (N = 46) into two groups, those who understood the speech (N = 23) and those who did not understand the speech (N = 23). This division is based on the participants’ answers to the third question of the questionnaire (Did you understand the speech? What were the individuals in the videos saying?).To investigate the effect of Language Comprehension (Understood vs Did not Understand), a repeated measures ANOVA was conducted including the Presence/Absence of an Eye Blink and the Orientation as within-subject factors and Language Comprehension as a between-subject factor on participants’ $d^\prime$ values. The results revealed a main effect of Language Comprehension on $d^\prime$ (F(1, 44) = 4.527, p = 0.039, $\eta$^2^ = 0.042). Participants who did not understand the speech (M = 0.625, SD = 0.541) had significantly lower sensitivity than participants who understood the speech (M = 0.369, SD = 0.202) (see Supplementary Fig. 1). We also investigated the effect of Language Comprehension based on C values. The results showed that Language Comprehension had no significant effect on participants’ C values (F(1, 44) = 2.762, p = 0.104, $\eta$^2^ = 0.022).

Further, we explored whether there was any relation between Language Comprehension and participants’ reaction times (see Supplementary Fig. 2). We performed a repeated-measures ANOVA using Language Comprehension as a between-subjects factor. The results showed a significant difference (F(1,44) = 5.355, p = 0.025, $\eta$^2^ = 0.088) between participants who did not understand the speech (M = 2.758, SD = 0.078) and the ones who understood the speech (M = 2.824, SD = 0.114). The mean score for C values of participants who did not understand the speech significantly differed from zero (t(44) = −0.158, p = 0.004), in contrast to those who understood the speech (t(44) = −0.045, p = 0.710).

### Eye tracking

To assess how well participants fixated the fixation cross throughout the trials in the different conditions, we analyzed their fixation patterns using the Labvanced data. We defined a square region with a side length of $4.2^{\circ }$ around fixation and quantified participants’ fixations within this region throughout the experiment. Overall, 91% of participants’ fixations during the trials were in this central region. Fixations outside these regions followed a clear pattern, with differences between upright and inverted face stimuli. Specifically, when faces were presented in an upright orientation, 12% (With Blink) and 13% (No Blink) of the participants’ fixations were directed towards the eyes (upper half of the faces). However, when the faces were inverted, only 4% (No Blink) and 5% (With Blink) of the participants’ fixations were directed towards the eyes. Also eye movements towards the mouth region differed between the two conditions. When the faces were presented in the upright orientation, only 1.6% (With Blink) and 1.7% (No Blink) of the participants’ fixations were directed towards the mouth region. When the faces were inverted, this percentage was increased: 4.4% (4.44% With Blink and 4.41% No Blink) of the participants’ fixations were directed towards the mouth region. Note that the eye movement direction to the eye and mouth regions (up or down) is confounded with the orientation of the face (upright or inverted). Overall, these findings show that the majority of participants’ fixations were within the central region, indicating successful fixation.

### Discussion (Experiment I)

In Experiment I, we examined whether participants’ sensitivity was affected by the Presence of an Eye Blink and the Orientation of the dynamic faces. To test this, we varied two factors: (1) an eye blink was either present or not; (2) the face was presented either upright or inverted. We measured sensitivity to discriminate whether the dynamic face movement was played forward or backward. To quantify the sensitivity, we calculated $d^\prime$ and C values, and measured RTs. Both for $d^\prime$ and C values, we observed significant main effects of the Presence/Absence of an Eye Blink and the Orientation of the face.

Sensitivity was higher in the “With Blink” compared to the “No Blink” condition and with “Upright” compared to “Inverted” faces. These results suggest that participants used the temporal information available in eye blinks to perform the task, with eye blinks aiding in determining whether videos were played forward or backward. The effect of orientation suggests that temporal order information can be extracted more effectively when faces are presented upright compared to inverted. Importantly, in all conditions, except for the “No Blink-Inverted” condition, participants’ sensitivity was above chance. The RT results showed that higher sensitivity in the “With Blink” compared to the “No Blink” condition was not due to a speed-accuracy tradeoff: Participants were not only more sensitive to timeline order when eye blinks were present, but they were also faster.

The observed patterns in participants’ C values shed light on the impact of Orientation on response biases when judging the Temporal Order. The significantly lower C values in the “Upright” condition compared to the “Inverted” show that participants were more likely to respond “forward” when faces were presented upright compared to inverted, possibly indicating a confusion of the orientations of the faces with the temporal order (see also discussion below). The results further show that in both "Upright" conditions, the C values were significantly lower than zero, suggesting a general bias to respond “forward” when the faces were upright. Specifically, both conditions, with and without a blink, yielded significantly negative mean C values. However, in the "Inverted" conditions, there was no significant deviation of C values from zero, suggesting no bias (regardless of the presence of an eye blink). 

Half of the participants understood the content of the (muted) speech. Compared to participants who did not understand the content, the sensitivity of participants who did understand the content was higher, suggesting that extracting meaning from the mouth and lip movement could be used to discriminate between the two different temporal orders of the videos. Alternatively, participants who performed well overall also understood the content of the speech without the latter causing the former. Experimental manipulations of speech comprehension will be able to distinguish between these two possibilities. The reaction time results show again that there was no speed-accuracy tradeoff: When participants understood the speech, they were also faster to respond. The results suggest that Language Comprehension helped participants to discriminate the temporal order of the videos.

The analysis of the eye tracking data revealed that the large majority of the participants’ fixations were within the defined central region. The observed differences in fixations outside this central region between upright and inverted face stimuli suggest a notable impact of stimulus orientation on participants’ gaze behavior. Specifically, when faces were presented upright, a significant proportion of participants’ fixations were directed towards the eyes regardless of whether faces were presented with or without a blink. This aligns with existing literature suggesting a natural tendency to prioritize eye contact for social cues^40^. In contrast, the participants’ fixations of the eyes were strongly reduced in the Inverted compared to the Upright condition, indicating a potential disruption in the typical gaze patterns associated with natural viewing conditions. Moreover, in the Upright condition, very few participants’ fixations were towards the mouth region regardless of the presence or absence of an eye blink. In the Inverted condition the ratio of fixating the eyes and mouth region was very similar.

## Experiment II: upright and inverted faces in different blocks

In Experiment I, biases (C values) differed between the “Upright” and “Inverted” conditions: Participants reported that the target was forward more often in the ”Upright” condition than in the “Inverted” condition. This bias difference may well have been driven by the tendency to report “forward” when the face was “Upright”, and “backward” when the face was “Inverted”. To control for this bias, we presented the “Upright” and “Inverted”conditions in separate blocks in Experiment II.

In total, each participant completed 432 trials (16 trials in the training session and 416 trials in the main experiment, 208 in each of the two blocks; i.e., twice the number of trials in Experiment I to increase reliability). The order of blocks (Upright first and Inverted second; Inverted first and Upright second) was counterbalanced across subjects. As in Experiment I, participants completed a training session, the main experiment and the questionnaire. During the training session and the main experiment, participants were asked to fixate the fixation cross. After each stimulus, the fixation cross remained on a black screen. Overall, the experiment lasted approximately 60 minutes, encompassing the training session, the main experiment, and the questionnaire.

### Participants

84 participants (52 females, 28 males, mean age: 21.63) participated in the study in exchange for course credits. Four of the participants were excluded due to misinterpretation of the task. The majority of the participants (85%) were Turkish native speakers. The rest of the participants spoke different levels of Turkish (4% very well; 6% a little; and 5% did not understand any Turkish). Participants who were not native Turkish speakers were excluded to reduce linguistic disparities as in Experiment I. Of the remaining 68 Turkish speakers, 7 were removed as all $d^\prime$ values were negative or because a negative $d^\prime$ value was the highest absolute value of all conditions. We included 61 participants (39 females, 22 males: M = 21.31, SD = 1.42; M= 21.68, SD = 1.52) in the further analysis of the data ($d^\prime$, C and RT). As in Experiment I, one participant was excluded from the eye tracking analysis due to missing eye tracking data.

## Results

The same statistical procedure is carried out for analyzing the data of Experiment II.

### Sensitivity and criterion ($d^\prime$ and C values)

A repeated-measures analysis of variance (ANOVA) was conducted to examine the effects of the Presence/Absence of an Eye Blink (“With Blink” vs “No Blink”) and the Orientation (“Upright” vs “Inverted”) on participants’ sensitivity to the Temporal Order measured by $d^\prime$ values. The main effect of the Presence/Absence of an Eye Blink was significant (F(1, 60) = 46.326, p $<0.001$, $\eta$^2^ = 0.258). The main effect of the Orientation was also significant (F(1, 60) = 56.391, p $<0.00$, $\eta$^2^ = 0.137). There was no significant interaction effect between the Presence/Absence of an Eye Blink and the Orientation. Participants showed better performance when the blink information was present: “With Blink” (M = 1.091, SD = 0.777) to “No Blink” (M = 0.393, SD = 0.343). Additionally, higher sensitivity was observed when the Orientation was “Upright” (M = 0.996, SD = 0.544) compared to “Inverted” (M = 0.487, SD = 0.494). Participants had significantly higher sensitvity in the “With Blink-Upright” condition than in the “With Blink-Inverted” condition (Mdif = 0.630, 95% CI [0.384, 0.875], p $<0.001$) and in the “No Blink-Upright” condition than in the “No Blink-Inverted” condition (Mdif = 0.388 95% CI [0.142, 0.633], p $<0.001$). Comparing the marginal means to zero revealed that sensitivity was above zero in all four conditions: “With Blink-Upright” (M = 1.405, 95% CI [1.229, 1.582], p $<0.001$), “With Blink-Inverted” (M = 0.776, 95% CI [0.600, 0.952], p $<0.001$), “No Blink-Upright”(M = 0.410, 95% CI [0.410, 0.763], p $<0.001$], “No Blink-Inverted” (M = 0.199, 95% CI [0.022, 0.375], p = 0.027).

**Figure 5:**
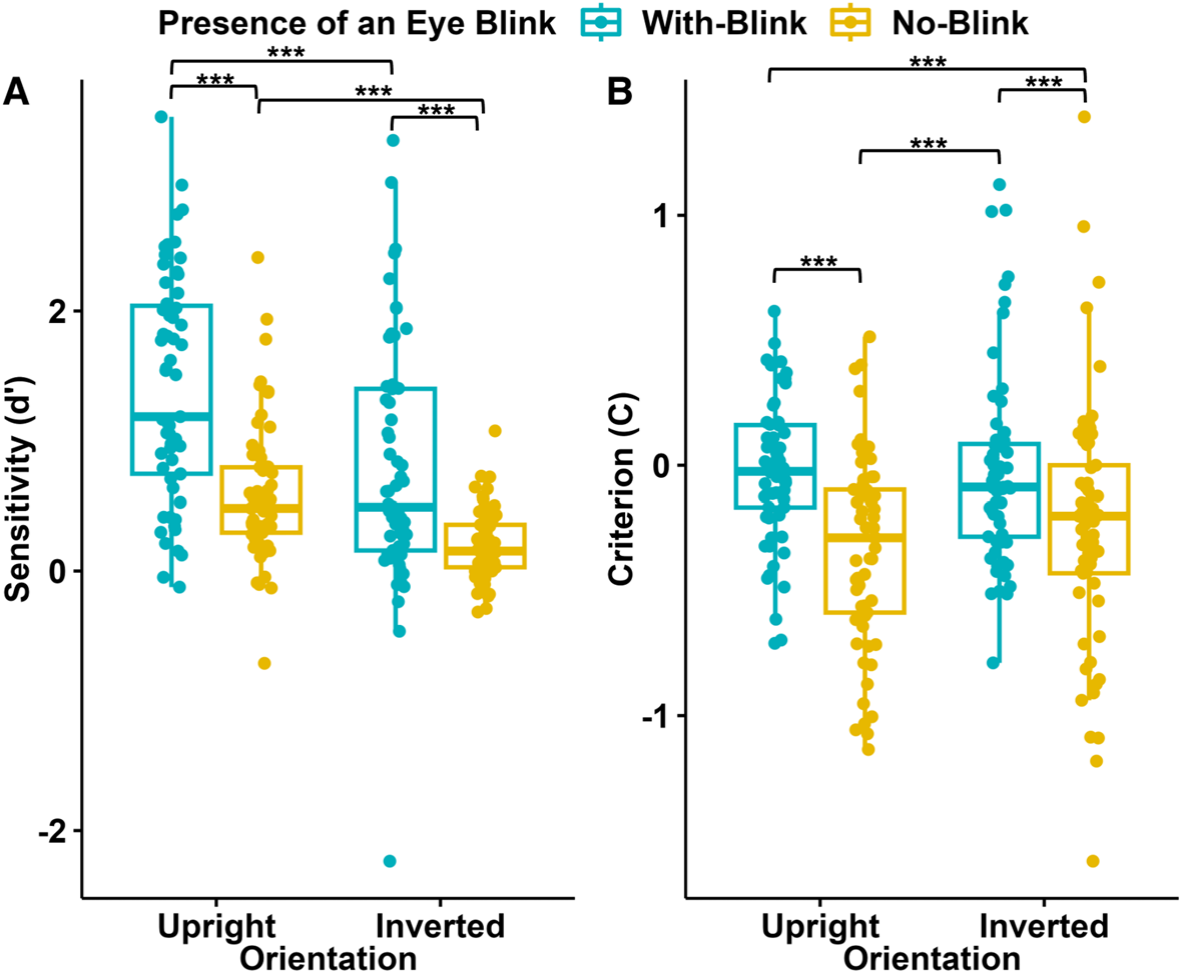
Sensitivity ($d^\prime$) and criterion (**C**) results (N = 61) of Experiment II. Blue data points represent the responses to the stimuli with eye blinks, and yellow data points represent responses to the stimuli without eye blinks; each for upright and inverted orientations of the faces. The y-axis represents $d^\prime$ (**A**) and C values (**B**). The 75th percentile is denoted by the upper hinge, and the lower hinge corresponds to the 25th percentile. Whiskers extend to values that lie within 1.5 times the interquartile range (IQR).

A repeated-measures ANOVA was conducted to examine the effects of the Presence/Absence of an Eye Blink and the Orientation on participants’ C values. The main effect of Presence/Absence of an Eye Blink was significant (F(1, 60) = 30.186, p $<0.001$, $\eta$^2^ = 0.198). Participants’ C values were higher in the “With Blink” condition (M = $-0.021$, SD = 0.305) than in the “No Blink” condition (M = $-0.286$, SD = 0.392). There was neither a significant main effect of the Orientation nor an interaction between the Presence/Absence of an Eye Blink and Orientation (Fig. 5).

### Reaction time (RT)

**Figure 6:**
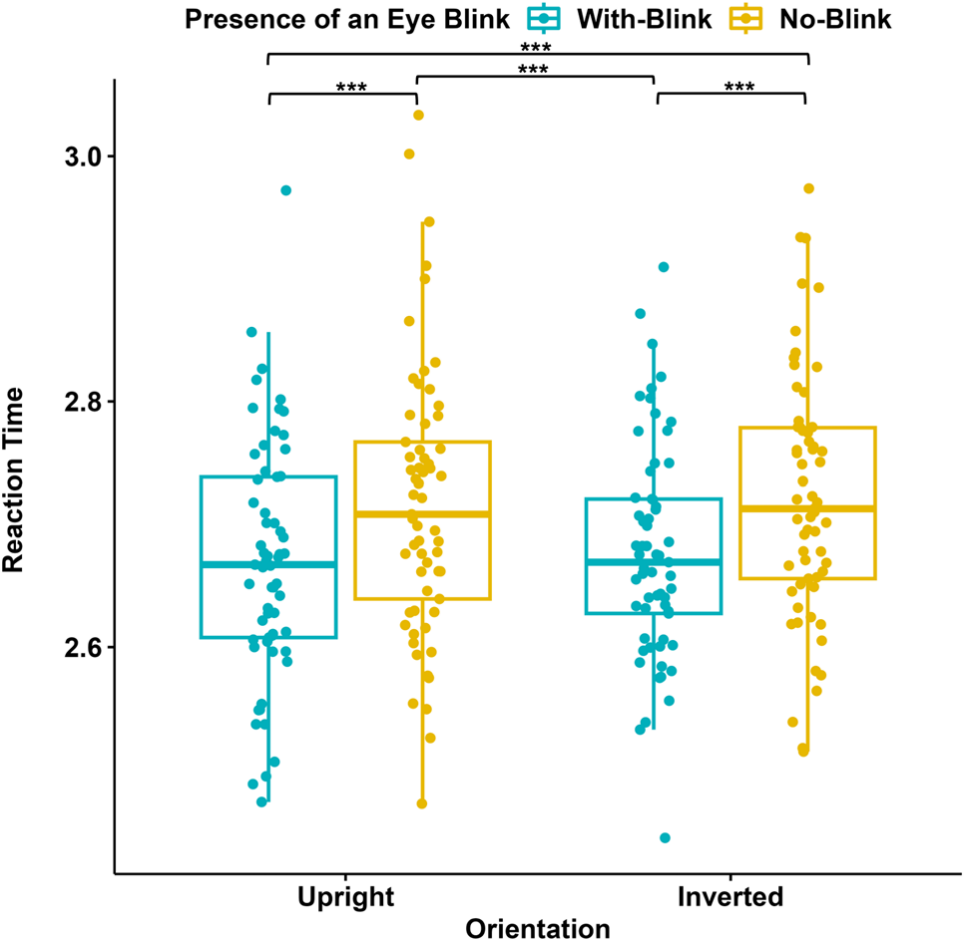
Reaction times (in seconds) in Experiment II (N = 61). Blue data points represent RTs for stimuli with eye blinks, and yellow data points represent RTs for stimuli without eye blinks; each for upright and inverted orientations of the faces. The y-axis represents RTs (log 10), while the x-axis represents the Orientation of the displayed stimuli. The 75th percentile is denoted by the upper hinge, and the lower hinge corresponds to the 25th percentile. Whiskers extend to values that lie within 1.5 times the interquartile range (IQR).

The reaction time data were normalized with the same procedure as in Experiment I. Then the data (N = 61) was analyzed by a two-way repeated measures ANOVA. The results revealed that there was a main effect of the Presence/Absence of an Eye Blink (F(1,60) = 26.796, p $<.001$, $\eta$^2^ = 0.151), with faster RTs when the blink information was present (“With Blink”; M = 2.674, SD = 0.085) compared to when it was absent (“No Blink”; M = 2.719, SD = 0.096). There was no main effect of Orientation (F(1,60) = 0.401, p = 0.529, $\eta$^2^ = 0.003) and no interaction (F(1,60) = 0.107, p = 0.107, $\eta$^2^ = 0, 156) (Fig. 6).

### Language comprehension ($d^\prime$, criterion, RT)

To explore whether Language Comprehension played a role in participants’ sensitivity and bias, we again split participants into two groups: participants who understood the speech (N = 12) and participants who did not understand the speech (N = 49). An independent sample Welch’s t-test was conducted to compare the mean $d^\prime$ values of these groups across the four conditions: “With Blink-Upright”, “With Blink-Inverted”, “No Blink-Upright”, “No Blink-Inverted”. For the “With Blink-Upright” condition, Welch’s t-test revealed a significant difference, (t(20.391) = $-1.852$, p = 0.039). Participants who did not understand the speech showed significantly lower sensitivity (M = 1.052, SD = 0.699) than the participants who understood the speech (M = 1.492, SD = 0.876). Similarly, in the “No Blink-Upright” condition, results showed a significant difference, (t(24.348) = $-4.077$, p $<.001$). Participants who did not understand the speech showed significantly lower sensitivity (M = 0.177, SD = 0.349) than the participants who understood the speech (M = 0.687, SD = 0.517). In the “Blink-Inverted” condition, there was no significant difference between groups, (t(15.810) = 0.178, p = 0.569). Participants who did not understand the speech (M = 0.821, SD = 1.009) did not differ significantly from those who understood it (M = 0.765, SD = 0.922). In the “No Blink-Inverted” condition, the analysis revealed no significant difference between groups, (t(17.543) = $-1.241$, p = 0.115). Participants who did not understand the speech (M = 0.116, SD = 0.255) did not exhibit a significant difference in $d^\prime$ values compared to those who did understand it (M = 0.219, SD = 0.270) (see Supplementary Fig. 3).

Further, we tested whether Language Comprehension was related to participants’ bias. An independent sample Welch’s t-test was conducted to compare the mean C values across the four conditions. For the “With Blink-Upright” condition, Welch’s t-test revealed a significant difference, (t(19.713) = $-2.562$, p = 0.019). The mean C values of the participants who did not understand the speech were significantly lower (negative) (M = $-0.187$, SD = 0.237) than the ones who understood the speech (M = 0.018, SD = 0.287). In the “No Blink-Upright” condition, results showed no significant difference, (t(19.394) = $-0.683$, p = 0.502). In the “Blink-Inverted” condition, there was a significant difference between groups, (t(33.141) = $-3.035$, p = 0.005). The mean C values of participants who did not understand the speech (M = $-0.228$, SD = 0.215) were significantly lower (negative) than those who did understand it (M = 0.031, SD = 0.410). In the “No Blink-Inverted” condition, the analysis revealed no significant difference between groups, (t(27.021) = $-2.054$, p = 0.050).

Finally, we tested whether the Language Comprehension was related to participants’ reaction times. In the “Blink-Upright” condition, the Welch’s t-test revealed no significant difference between groups, (t(18.400) = $-1.237$, p = 0.884). Participants who did not understand the speech (M = 2.641, SD = 0.089) did not significantly differ in reaction time from those who understood (M = 2.677, SD = 0.099). Similarly, in the “Blink-Rotated” condition, there was no significant difference between groups, (t(17.352) = 0.902, p = 0.190). Participants who did not understand the speech (M = 2.698, SD = 0.086) did not significantly differ in reaction time from those who understood it (M = 2.672, SD = 0.089). In the “No-Blink-Upright” condition, the results showed no significant difference, (t(13.514) = $-0.606$, p = 0.723). Participants who did not understand the speech (M = 2.694, SD = 0.150) did not significantly differ in reaction time from those who understood the speech (M = 2.722, SD = 0.100). In the “No-Blink-Rotated” condition, there was no significant difference between groups, (t(15.101) = 0.358, p = 0.363). Participants who did not understand the speech (M = 2.732, SD = 0.116) did not significantly differ in reaction time from those who understood the speech (M = 2.719, SD = 0.099) (see Supplementary Fig. 4). Similar to the Experiment I, the mean score for C values of participants who did not understand the speech was significantly lower than zero (t(59) = −4.258, p < 0.001) in contrast to participants who understood the speech (t(59) = −0932, p = 0.710).

### Experiment type and block order ($d^\prime$ and C values)

We further investigated the impact of experimental design. To do that we compared Experiment I (randomized trials) and Experiment II (blocked order), by conducting a mixed ANOVA on $d^\prime$ and C values with experiment type used as a between-subject factor. According to the results, experiment type had a significant effect on the $d^\prime$ values of the participants (F(1, 105) = 8.230, p = 0.005, $\eta$^2^ = 0.027). Participants showed lower sensitivity in Experiment I (randomized trials) compared to Experiment II (blocked order) (Mdif = $-0.245$, 95% CI [$-0.414$, $-0.076$], p = .005). The analysis of C values did not reveal a significant effect of the experiment type (F(1, 105) = 0.978, p = 0.325, $\eta$^2^ = 0.004). Additionally, we measured the impact of the block order (upright first vs. inverted first) in Experiment II on $d^\prime$ and C values. The results showed no significant main effect of block order, neither for the $d^\prime$ (F(1, 59) = 0.089, p = 0.767, $\eta$^2^ = 0.4434) nor for the C values (F(1, 105) = 0.978, p = 0.325, $\eta$^2^ = 0.004).

### Eye tracking

We applied the same analysis as in Experiment I. Similar to Experiment I, in Experiment II our analysis uncovered a substantial disparity in eye movement patterns between upright and inverted face stimuli. Overall, 86% of participants’ fixations were in the central region (square region with a side length of $4.2^{\circ }$ around fixation). When faces were presented in the upright orientation (“With Blink” and “No Blink”), 22% of fixations were directed towards the eyes (i.e., outside of this central region). Conversely, when faces were inverted, only about 2% (1.9% With Blink, and 2% No Blink) of participants’ fixations were directed towards the eyes. Eye movements towards the mouth region also differed between the two Orientation conditions: In the Upright condition only 1.5% (both With Blink and No Blink) of the fixations were directed towards the mouth region (lower half of the faces). In the Inverted condition, 6% (both With Blink and No Blink) of the fixations were directed towards the mouth region.

### Discussion (Experiment II)

In Experiment II, we examined whether sensitivity differences -and in particular bias differences- found in Experiment I were related to the randomly interleaved presentation of upright and inverted faces. In particular, we sought to investigate if the stronger bias to report “forward” when upright faces were presented (and the comparatively larger bias to report “backward” when inverted faces were presented) reflected the mistaken reporting of face orientation instead of temporal order. In line with this hypothesis, C values did not differ between the “Upright” and “Inverted” conditions in Experiment II. In the “With Blink” condition, C values were close to zero. The inclination to report “forward” in the “No Blink” condition suggests that if no deviation from normal was detected, participants tended to report that the temporal order was forward. The pattern of sensitivity results was similar to the pattern in Experiment I: Sensitivity was higher with eye blinks compared to no eye blinks, and close to zero in the Inverted condition without eye blinks. As in Experiment I, sensitivity in the Inverted condition with eye blinks was again higher than in the Inverted condition without eye blinks, suggesting that eye blinks were used by participants to perform the task. Notably, sensitivity was higher than chance levels in all conditions except for the Inverted condition without an eye blink. The reaction time results revealed a significant impact of the Presence/Absence of an Eye Blink. Specifically, faster reaction times were observed when an Eye Blink was present compared to when it was absent.

As in Experiment I, we explored if there were any sensitivity, C, and RT differences between participants who understood the speech compared to participants who did not understand the speech. Overall, sensitivity was higher when participants understood the speech compared to when they did not understand the speech. In contrast to Experiment I, participants who understood the speech showed significantly higher sensitivity when the Orientation was “Upright” (“With Blink-Upright” and “No Blink-Upright”) compared to “Inverted” (“With Blink-Inverted” and “No Blink-Inverted”). In the “Inverted” condition, there was a trend for higher sensitivity when participants understood the speech compared to when they did not understand the speech. The examination of the relationship between Language Comprehension and participants’ bias showed that responses were (negatively) biased when participants did not understand the speech compared to when they understood the speech. This result suggests a bias to respond “forward" when not understanding the speech, possibly because “forward” was the preferred response when not detecting any deviations from “forward” (or “normal”). Unlike Experiment I, no significant reaction time differences were observed between understanding and not understanding the speech. Note that the ratio of participants who understood the speech was lower in Experiment II than in Experiment I (see Supplementary Fig. 3). The better language comprehension in Experiment I compared to Experiment II may be attributed to a strategy to explore the stimuli more thoroughly in Experiment I for any information that could help to perform the task. Higher stimulus variance in between trials might have rendered the task more difficult in Experiment I than Experiment II, leaving fewer ressources to develop a strategy.

In Experiment II, the examination of eye tracking data revealed highly similar fixation patterns as in Experiment I. The majority of fixations were within the predefined central region. Noteworthy patterns emerged when comparing fixations outside this central region between upright and inverted face stimuli, revealing a significant influence of stimulus orientation on participants’ fixations. In the upright condition, similar to Experiment I, a considerable number of participants directed their fixations towards the eyes, irrespective of the presence or absence of an eye blink, again aligning with existing literature emphasizing a natural inclination to prioritize the eye region for social cues^40^. Conversely, in the Inverted conditions, participants exhibited a reduced tendency to fixate on the eyes, suggesting a potential disruption in the typical gaze patterns associated with natural conditions. Moreover, as in Experiment I, both the Upright and Inverted conditions in Experiment II demonstrated few fixations towards the mouth region, regardless of the presence or absence of an eye blink.

## General discussion

Faces are a rich source of information, including major social cues that are often crucial for human communication^1–7,41^. Studies on face perception showed that faces are processed holistically^12,14,42–44^. The holistic processing of faces can be disrupted by the face-inversion effect, possibly because attention shifts to partial information coming from parts such as the eyes and lips^12,44,45^. Studies on face perception mostly relied on static images and examined holistic perception in the spatial domain. In real life, however, faces are highly dynamic, containing important temporal information. A number of studies conducted experiments with dynamic faces as stimuli^46^, investigating the processing of emotional facial expressions^8,47^. However, dynamic faces are a rich source of information also without emotional facial expressions, for example, when verbally communicating with neutral facial expressions (which occurs highly frequently in real life). Here, using dynamic videos of faces with neutral facial expressions enabled us to investigate the perception of facial dynamics beyond expressing emotions. In particular, we investigated the impact of face orientation (upright or inverted) and an eye blink (present or absent) on dynamic face processing by measuring sensitivity to the temporal order of face dynamics (forward or backward). In two experiments, we presented dynamic face videos either upright or inverted, and either with or without an eye blink. In Experiment I, all stimuli were presented in random order; in Experiment II, they were blocked by Orientation. Overall, the findings demonstrated that participants were more sensitive to the temporal order when the stimuli were presented upright compared to inverted, and when they included an eye blink compared to no eye blink.

The sensitivity in all conditions of Experiment I was relatively modest, with a sensitivity reaching the highest $d^\prime$ value (of close to 1) in the “With Blink-Upright” condition. However, sensitivity was above chance level ($d^\prime$> 0) in all conditions, except for the Inverted condition without eye blinks, indicating that participants could perform the task also without eye blinks (when the faces were upright) and when faces were inverted (only when an eye blink was present as well). The pattern of results for sensitivity in Experiment II was the same as in Experiment I: Sensitivity in the Upright condition was higher than in the Inverted condition, and in the With-Blink compared to the No-Blink condition. There was no interaction between Orientation and Presence/Absence of an Eye Blink. However, sensitivity was higher in Experiment II compared to Experiment I. We suggest that this result was due to the different designs: Fully randomized in Experiment I and blocked by Orientation in Experiment II. The high inter-trial variance, with frequent salient stimulus changes in between trials (from Upright to Inverted, and Inverted to Upright) in Experiment I may have interfered with performing the task. The absence of this source of variance in Experiment II, by contrast, could have led to better concentration on the relevant features to perform the task. The interpretation that superior sensitivity in Experiment II is due to the two different procedures, instead of, for example, group differences, is supported by the different patterns of criterion results (see below).

Higher sensitivity in the Upright condition compared to the Inverted condition replicates many findings in face perception research^42,48^. Extensive research has shown that observers’ recognition accuracy^49–51^ and efficiency is superior when perceiving faces presented in their canonical upright orientation compared to inverted orientation^44,45,52^. This effect highlights the specialized neural mechanisms dedicated to upright face processing, suggesting that the brain employs configural and holistic processing strategies optimized for upright faces^42^. Here, we show that the advantage for upright faces, usually shown with static faces for tasks such as face recognition^53^, emotion perception^54^, the perception of sex^55^ and age^56^ also holds for the perception of dynamic faces when judging their temporal order. Importantly, the advantage for Upright compared to Inverted faces was observed when an eye blink was present and when no eye blink was present. As there were two main sources based on which temporal order judgments could be made (eye blinks and mouth movements), these results suggest that both contributed more strongly to discriminating the temporal order when faces were presented upright compared to inverted. In the condition without eye blink, mouth movements were the principal source for the temporal order judgments (in addition to less salient cues, such as nostril movement during breathing and subtle facial movements other than by the mouth and eyes). The lower sensitivity in this condition compared to the condition with eye blink suggests either that eye blinks were a more reliable source to determine the temporal order, or that there was an advantage when having two sources (an eye blink and mouth movements) to perform the task (there was no condition with eye blink but without mouth movements which should be investigated in future studies). However, in the conditions with an eye blink, there was a single eye blink at varying times during the 3-second videos, while mouth movements were visible throughout the entire trial. Having two highly different, but both useful, sources available allows for different strategies to perform the task. For example, observers could mainly (or exclusively) attend to only one of the sources and use the information from that single source. Alternatively, they could attend to both sources and integrate the extracted information to make their judgment, or attend to both sources and respond in line with the source that provides stronger evidence for one or the other response within a given trial. To get a better idea of what strategies observers used in the current study, we asked them at the end of the experiment based on what information they made their decision to report forward or backward motion (see Supplementary Tables 1 and 2). In Experiment I, the large majority of participants reported to have based their decisions to indicate “forward” on the mouth. Also, a clear majority reported to have used the mouth to determine “backward” responses; however, several participants also reported to have used eye blinks or both mouth movements and eye blinks to make their decisions. Interestingly, the pattern of results was different in Experiment II where the majority of participants reported using information from the mouth for “forward” decisions and eye blinks for “backward” decisions. These results may indicate that participants used mouth movements as a default to perform the task, and eye blinks when they noticed that the eye blink looked somewhat “unnatural”. However, they also seem to suggest, as mentioned above, that different strategies can be used to determine the temporal order of dynamic faces. Future studies that systematically vary the information available in the different sources to determine temporal order will show how flexible observers can use different strategies to maximize performance. These studies are also important to quantify to what extent partial facial cues are subject to inversion effects when no other information is available and when not embedded in a face. For example, explicitly telling observers that the task is to determine the temporal order based on mouth movements or eye blinks alone, and fixing the other cue to be uninformative can determine to what extent each of these cues is subject to inversion effects. Additionally, different partial cues can be presented in isolation while informing observers about the presented orientation. A cost to report the temporal order with inverted cues compared to the canonical orientation could be expected, similar to the inversion effect found here for mouth movements and eye blinks.

In Experiment I, participants’ RTs were faster when the orientation was upright compared to inverted. This finding is consistent with previous research on face perception, which suggests that disrupting the holistic view of a face can increase the time needed for processing^57,58^. However, in Experiment II, there was no difference in RTs between the Upright and Inverted conditions. The difference between upright and inverted faces in Experiment I could be due to a general advantage with upright compared to inverted faces that only manifested when there was a switching cost, when having to attend to upright and inverted faces randomly interleaved in the same block. RTs were faster in both experiments when there was an eye blink compared to when there was no eye blink. This RT difference suggests that the presence of an eye blink facilitated participants’ temporal order judgments. Importantly, there was no speed-accuracy tradeoff in either of the two experiments.

The orientation of the faces and the Presence/Absence of an eye blink also played a role in the criterion results (C values). In Experiment I, there was a strong difference between the Upright and the Inverted condition: When faces were presented upright, participants tended to report “forward” more often than when faces were presented inverted. This difference in criterion shows that responses to the temporal order were partly influenced by the orientation of the face. A possible explanation of this response pattern might be that participants associated ‘upright’ responses to some extent with the ‘forward’ or ‘normal’ temporal order, and ‘inverted’ responses with the ‘backward’ or ‘not normal’ temporal order. To test this explanation, we conducted Experiment II where the Upright and Inverted conditions were presented in separate blocks. If erroneous associations between spatial (upright and inverted) and temporal (forward and backward) factors were underlying the criterion difference, the effect should be reduced when presenting the two orientations in different blocks as -in this case- all trials (within a block) are of the same orientation, and using different orientations to respond regarding the temporal order would be less likely. In line with this assumption, there was no criterion difference between the Upright and Inverted conditions in Experiment II. The criterion in Experiment II also depended on the Presence/Absence of Eye Blinks: When an eye blink was present, C values were closer to zero compared to when no eye blink was present, showing that there was little or no bias when discriminating the temporal order with the possibility to use eye blink information. In the absence of eye blinks, negative C values suggest a tendency among participants to report the temporal order as forward when lacking the partial cue eye blink.

At the end of the experiments, participants were asked if they had understood the speech (and if so, what was the content of the speech). This question about language comprehension was used to explore whether there was any relation between deciphering what was said in the face videos and sensitivity (as well as criterion and RT). A significant number of participants in Experiment I (23 out of 46) understood the speech. Interestingly, the proportion of participants who understood the speech was much smaller in Experiment II (only 12 out of 61). Nevertheless, in both Experiments, the sensitivity of participants who did understand the content was higher compared to participants who did not understand the content, suggesting that extracting meaning from the mouth and lip movement could be used to discriminate between the two different temporal orders of the videos. Alternatively, participants who performed well overall also understood the content of the speech without the latter causing the former. Experimental manipulations of (the possibility for) language comprehension will be able to distinguish between these two possibilities. The reaction time results show again that there was no speed-accuracy tradeoff: When participants understood the speech, they were also faster to respond. The RT results similarly suggest that Language Comprehension helped participants to discriminate the temporal order of the videos. Note that per participant there was only a single data point -either participants understood the speech (at any or multiple points during the experiment) or they did not. The reasoning for including this question was that understanding the speech in a given trial should be highly correlated with correctly reporting the temporal order: Understanding the speech implies correctly extracting the temporal order. Why there was such a large difference regarding the proportion of participants understanding the speech and not understanding the speech in Experiments I and II is unclear. One possible reason for the difference is that participants in Experiment I, where the temporal order task seemed to have been more difficult (as shown by the lower sensitivity), were exploring the stimuli more thoroughly for any information that could have helped them to perform the task. Importantly, as we do not know in which trials participants understood the speech and whether they noticed that it was the same speech in all trials, it is not possible to infer the number of trials in which discerning the content of the speech was used by participants to discriminate the temporal order.

Comparing Experiments I and II, revealed that participants showed lower sensitivity in Experiment I compared to Experiment II, indicating that participants’ sensitivity to temporal order was influenced by the random vs blocked presentation of the Orientation of the faces. The higher sensitivity in Experiment II may have been due to the lower inter-trial variance and, correspondingly, better focus on the task at hand. Importantly, in addition to higher sensitivity, C values were closer to zero, suggesting that the relatively strong bias in Experiment I was at least partly due to task confusions, reporting the orientation of the face instead of its temporal order. Hence, there is evidence for our initial interpretation that the randomly interleaved presentation of the two different orientations (upright and inverted) in Experiment I caused the bias to report backward temporal order when presented with inverted faces. As the goal is usually to avoid strongly biased responses, it seems advisable to favor the blocked design as in our Experiment II when erroneous responses to task-irrelevant dimensions can be expected.

Although participants were instructed to fixate on the fixation cross throughout each trial, eye movements were made, both in the direction of the eye regions and the mouth regions. In both experiments, there was a clear difference between the Upright and Inverted conditions. When faces were presented upright, much more eye movements were directed towards the eyes (about 12.5% in Experiment I and about 22% in Experiment II) compared to the mouth (about 1.5% in both experiments). In the Inverted condition, there were overall fewer eye movements away from fixation, and they were similarly frequent towards the eye and the mouth region in Experiment I (about 4.5%) and more frequent to the mouth region in Experiment II (about 6% to the mouth vs 2% to the eyes). (The Presence/Absence of Eye blinks did not modulate eye movement patterns.) These results support the notion that participants preferentially used information from the eyes in the Upright condition, but not in the Inverted condition. However, as these are “erroneous” eye movements, that is, they were performed despite different experimental instructions, and only a relatively small number of trials contained such erroneous eye movements, we cannot infer with certainty that similar attentional allocations occurred in trials when fixation was kept in the central region (on the fixation cross).

Our study is a precursor to future studies that will shed more light on the way temporal information is integrated in dynamic face perception. For example, parametrically varying the different partial cues can help to better understand what information is key to determining whether the temporal order is forward (‘normal’) or backward. In particular, different temporal segments of mouth movements and eye blinks can be investigated separately. In the present study, characteristic mouth movements during different segments of speech -for example, characteristic mouth movement patterns for different words and phonemes- presumably contain different levels of information that can be used to perform the temporal order task. Similarly, eye blinks and other facial movements vary strongly regarding their specific dynamics and presumably serve to different degrees as diagnostic criteria to evaluate temporal orders. An initial next step to better understand the exact contributions of different partial cues could be -as suggested above- to separately present partial cues (for example, only the eye region of faces with different segments of eye blinks, and different phonemes articulated with natural mouth movements), and investigate how these partial cues are combined when presented within a single face stimulus.

In conclusion, our findings show how human observers use different information sources, such as mouth movements and eye blinks to make temporal order judgments. Eye blinks seem to be particularly diagnostic to determine the temporal order of dynamic faces. However, the tendency to preferably attend to -and look at- eyes may play a role as well. We suggest that partial information from different sources is flexibly used by observers to maximize performance in the task at hand. We revealed the well-known face-inversion effect with dynamic face stimuli, showing that this typically spatial effect also occurs in the temporal domain. Our results also show that the dynamics of (neutral) faces during speech contain diagnostic information beyond emotional expressions that can be used to determine their temporal order. Finally, we suggest that holistic representations of temporal order are an important research topic to better understand how the human brain integrates and represents temporally distinct patterns that might well be the temporal equivalent to spatial Gestalts.

### Supplementary Information

Supplementary Information.

## Data Availability

The datasets generated and/or analyzed during the current study are available in the DynamicFaceBehavioral repository, https://github.com/Alp-Visual-Neuroscience-Lab/DynamicFaceBehavioral.
