# Author Correction: The effect of processing partial information in dynamic face perception

**DOI:** 10.1038/s41598-024-63709-1

**Published:** 2024-06-06

**Authors:** Nihan Alp, Gülce Lale, Ceren Saglam, Bilge Sayim

**Affiliations:** 1 Psychology, Sabanci University, Istanbul, Türkiye; 2 Graduate School of Systemic Neurosciences, Ludwig-Maximilians-Universität München, Munich, Germany; 3 Department of General Psychology, University of Padua, Padova, Italy; 4 Univ. Lille, CNRS, UMR 9193, SCALab – Sciences Cognitives et Sciences Affectives, F – 59000, Lille, France

Correction to: *Scientific Reports* 10.1038/s41598-024-58605-7, published online 29 April 2024

The original version of this Article contained errors in Figures 4 and 6, where y-axis label “Reaction Time” was incorrectly given as “Reaction Time (log10)”. The original Figures 4 and 6 and accompanying legends appear below.

**Figure 4:**
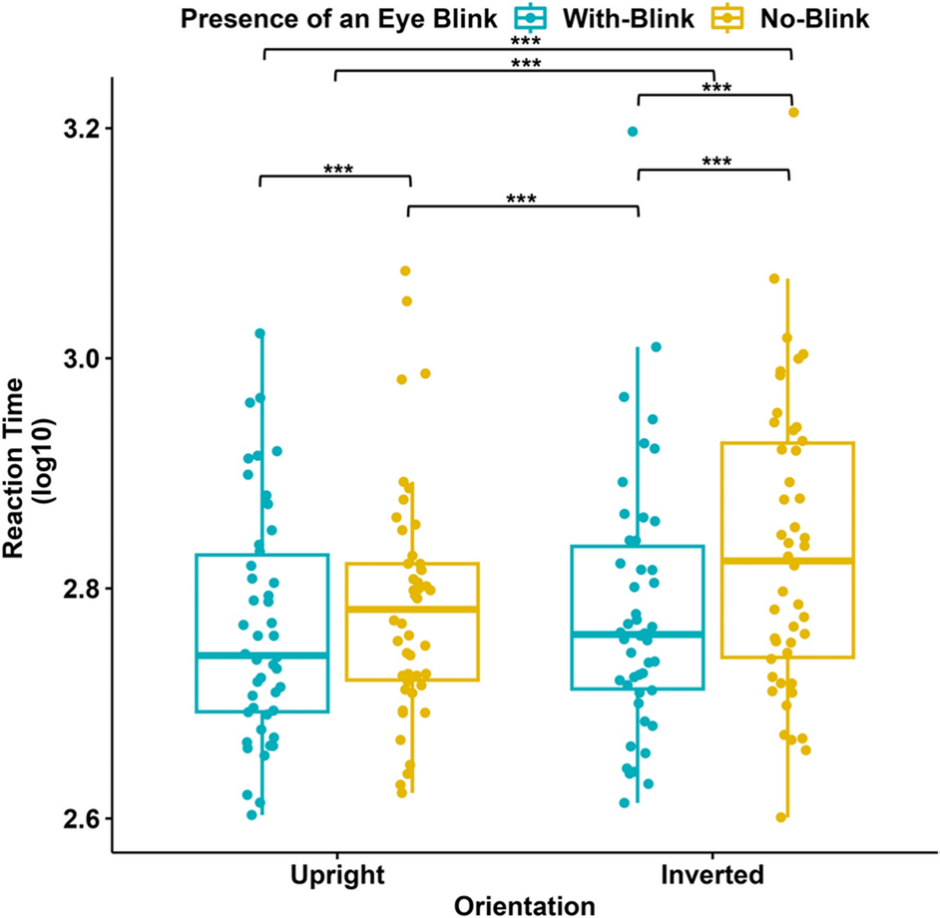
Reaction times (in seconds) in Experiment I (N = 46). Blue data points represent RTs for stimuli with eye blinks, and yellow data points represent RTs for stimuli without eye blinks; each for upright and inverted orientations of the faces. The y-axis represents RTs (log10). The 75th percentile is denoted by the upper hinge, and the lower hinge corresponds to the 25th percentile. Whiskers extend to values that lie within 1.5 times the interquartile range (IQR).

**Figure 6:**
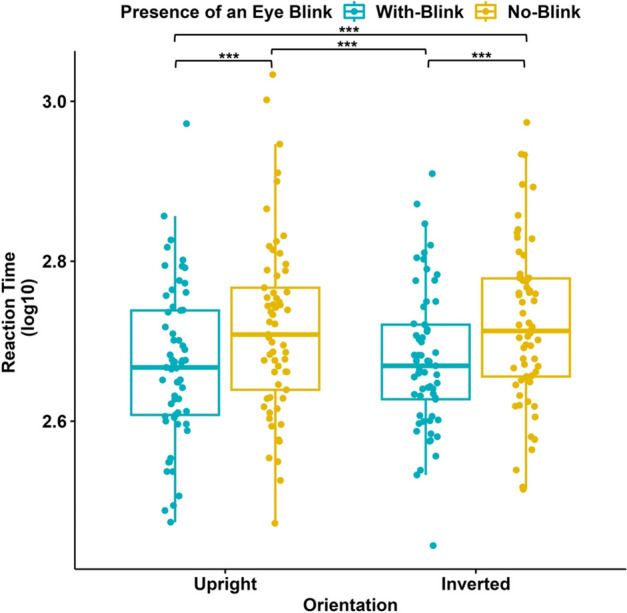
Reaction times (in seconds) in Experiment II (N = 61). Blue data points represent RTs for stimuli with eye blinks, and yellow data points represent RTs for stimuli without eye blinks; each for upright and inverted orientations of the faces. The y-axis represents RTs (log 10), while the x-axis represents the Orientation of the displayed stimuli. The 75th percentile is denoted by the upper hinge, and the lower hinge corresponds to the 25th percentile. Whiskers extend to values that lie within 1.5 times the interquartile range (IQR).

The original Article has been corrected.

