# Supplementary material for: The effect of processing partial information in dynamic face perception
Source: Sci Rep. 2024 Apr 29;14:9794. doi: 10.1038/s41598-024-58605-7 (PMC11059172; doi:10.1038/s41598-024-58605-7)
Supplement: Supplementary file 1 — Supplementary Information. [file 41598_2024_58605_MOESM1_ESM.pdf]

Questionnaire

Participants were presented with the following questions both in English and Turkish:

1. How did you decide if a video was in sequence? (Videonun normal yönde olduğuna nasıl karar verdiniz?)
2. How did you decide if a video was in reverse? (Videonun ters yönde olduğuna nasıl karar verdiniz?)
3. Did you understand the speech?What were the individuals in the videos saying? (Videodaki konuşmaları anladınız mı? Videolardaki kişiler ne hakkında konuşuyordu?)

Supplementary Table 1

"Other" responses from the participants (N=7) to the Q1 (How did you decide if the video was sequence?) were as follows: *Note that we only corrected small typos of participants such as "anythong" was corrected to "anything"

1. "there is not anything odd"
2. "Mimiklerine bakarak anlamaya çalıştım"(I tried to understand by looking at the facial expressions)
3. "There seems to be a fluidity when it is in sequence"
4. "genel olarak sanki biri benle konuşsa neler yapardı diye karar vermeye çalıştım ve çene ve yüz hareketlerine çok dikkat ettim. Normal konuşan birinden farklı hareketler yapmıyorsa normaldir dedim"(In general, I tried to decide as if someone were talking to me, considering what they would do, and I paid close attention to jaw and facial movements. If the person did not exhibit different movements from a regular speaker, I considered it as normal.)
5. "Nefes alırken burun delikleri küçülüp tekrar eski haline geldi" (The nostrils shrink and return to their normal state while breathing)
6. "Mimiklerinden" (Mimics)
7. "Yüzlerin herhangi bir tarafında alışılmadık bir hareket görmeyince" (When there is no unusual movement on any part of the faces)

"Other" responses from the participants (N=4) to the Q2 (How did you decide if the video was reverse?) were as follows:

1. "Mimiklerine bakarak anlamaya çalıştım" (I tried to understand by looking at the facial expressions)
2. "The motions do not feel smooth and natural"
3. "Nefes alırken burun delikleri büyüyüp tekrar eski haline geldi" (While breathing, the nostrils enlarged and returned to their original state)
4. "Boğumlamalar ve burun etrafındaki yanak çizgilerinde anormallik vardı." (proper articulation and there were abnormalities in the cheek lines around the nose)

Table 1. Experiment 1 Questionnaire Results

	Questions	
**Answer**	How did you decide if the video was sequence? (Q1)	How did you decide if a video was reverse?(Q2)
**Mouth**	32	24
**Blink**	2	11
**Both**	5	7
**Other**	7	4
Did you understand the speech? (Q3)	Yes	No
Number of Participants	23	23

Note. The numbers of answers in these tables are calculated from 46 participants' responses.

Supplementary Table 2

"Other" responses from the participants (N=4) to the Q1(How did you decide if the video was sequence?) were as follows:

1. "It looked natural"
2. "Bazen söylediklerini anlamaya çalıştım, yüz hareketleri doğal geliyorsa normal olduğunu düşündüm."
3. "yanakların yukarı yönlü hareketinden"
4. "People had normal face gestures"

"Other" responses from the participants (N=5) to the Q2 (How did you decide if the video was reverse?) were as follows:

1. "Mimiklerden" (Mimics)
2. "başlarını genelde insanlar konuşurken aşağı döndürürler, geri olanlarda tam tersi olduğunu düşündüm. Çene hareketleri ve yutkunmaları da ters geldi" (They generally turn their heads down when people talk, and in the reversed ones, I thought it was the opposite. Jaw movements and swallowing seemed reversed too)
3. "yüz hareketlerinden" (facial movements)
4. "mimikler ve hareketler normale göre farklı ise ters yönde olduğunu düşündüm."(I thought it was in reverse if facial expressions and movements were different from normal.)
5. "Normalde baktıklarımın terslerine baktım"(I looked at the opposite of what I normally look at)

Table 2. Experiment 2 Questionnaire Results

	Questions	
**Answer**	How did you decide if the video was sequence? (Q1)	How did you decide if a video was reverse?(Q2)
**Mouth**	34	19
**Blink**	7	28
**Both**	16	9
**Other**	4	5
Did you understand the speech? (Q3)	Yes	No
Number of Participants	49	12

Note. The numbers of answers in these tables are calculated from 61 participants' responses.

Figure 1: Language Comprehension: Sensitivity and criterion (Experiment I)

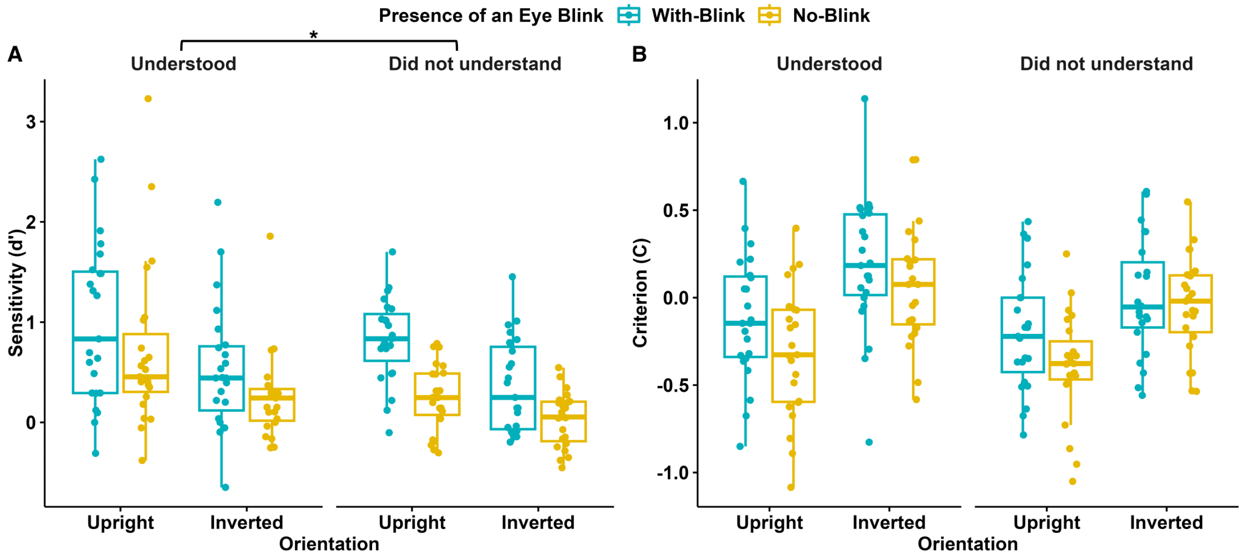

Figure 1. Sensitivity (d') and criterion results (N = 46) of Experiment I are shown in panel A and B, respectively. The plots are separated by Language Comprehension (Understood, N = 23; did not understand, N = 23). Stimuli either contained (blue data points) or did not contain (yellow data points) an eye blink. The orientation of the faces was either upright or inverted. The y-axis represents d' (A) and C values (B). Upper and lower hinges correspond to the 75th and 25th percentiles, and whiskers extend to values within 1.5 times the interquartile range (IQR).

Figure 2: Language Comprehension: RT (Experiment I)

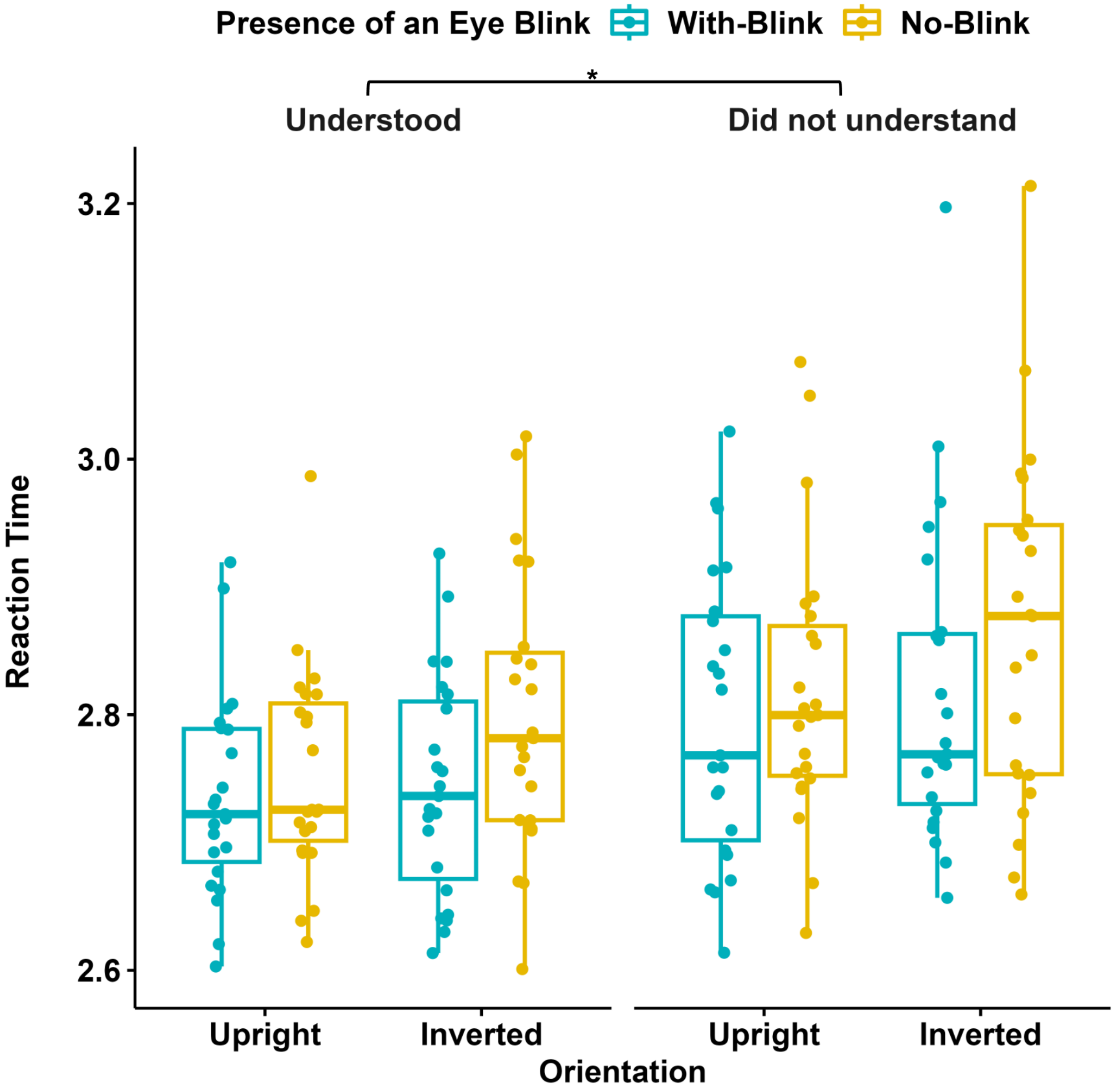

Figure 2. Reaction times (RTs; log10) in seconds of participants (N = 46) in Experiment I based on Language Comprehension (Understood N = 23; did not understand N = 23). Blue data points represent RTs for stimuli with eye blinks, and yellow data points represent RTs for stimuli without eye blinks, each for upright and inverted orientations of the faces. The y-axis represents RTs; log10 (a for overall) and c values (b for divided based on language comprehension). The 75th percentile is denoted by the upper hinge, and the lower hinge corresponds to the 25th percentile. Whiskers extend to values that lie within 1.5 times the interquartile range (IQR).

Figure 3: Language Comprehension: Sensitivity and Criterion (Experiment II)

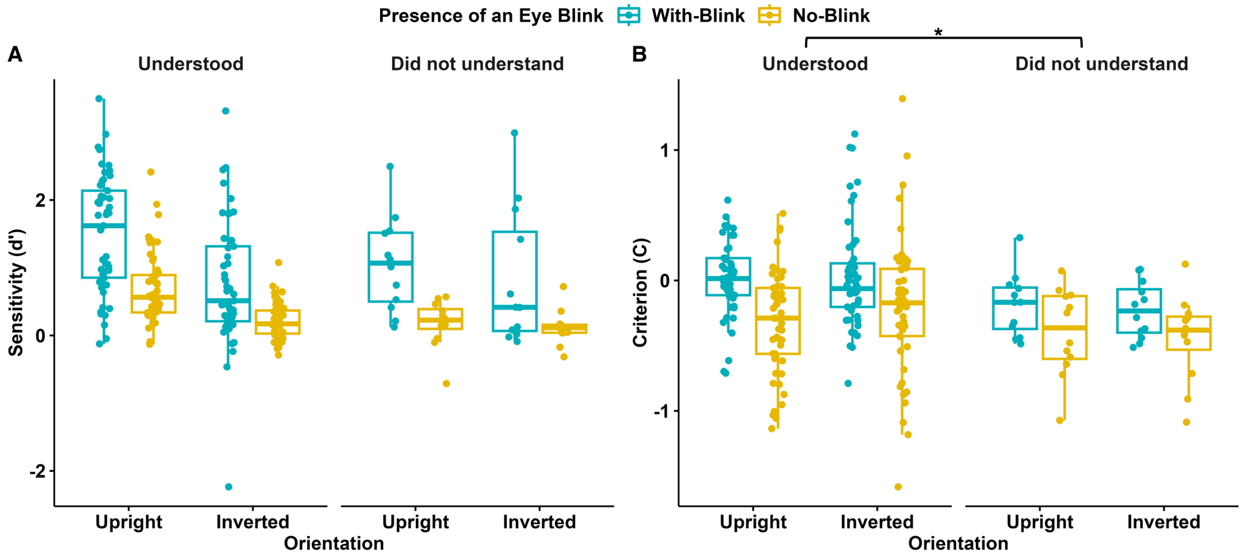

Figure 3. Sensitivity (d') in A and criterion results in B (N = 61) of Experiment II are displayed grouped by Language Comprehension of the participants. 12 participants understood the speech, while 49 participants did not understand the speech. Stimuli either contained (blue data points) or did not contain (yellow data points) an eye blink. The orientation of the faces was either upright or inverted. The y-axis represents d' (A) and c values (B). The y-axis represents d' (A) and c values (B). The upper and lower hinges represent the 75th and 25th percentiles, respectively, while the whiskers stretch to values that are 1.5 times the interquartile range (IQR).

Figure 4: Language Comprehension: RT (Experiment II)

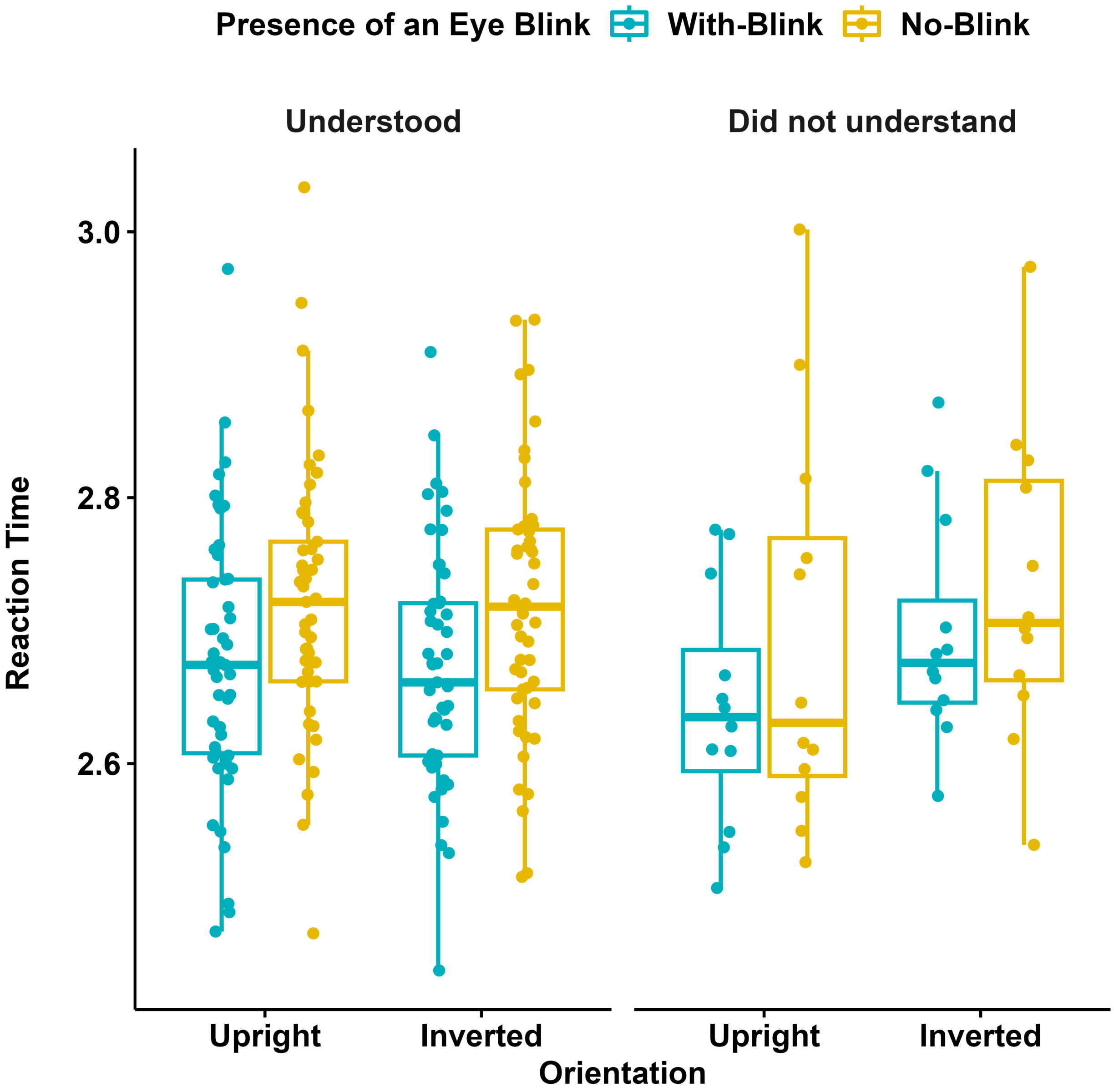

Figure 4. Reaction times (RTs; log10) in seconds of participants (N = 61) in Experiment II based on language comprehension. 12 participants understood the speech, while 49 participants did not understand the speech. Blue data points represent RTs for stimuli with eye blinks, and yellow data points represent RTs for stimuli without eye blinks; each for upright and inverted orientations of the faces. The y-axis represents RTs; log10. The 75th percentile is denoted by the upper hinge, and the lower hinge corresponds to the 25th percentile. Whiskers extend to values that lie within 1.5 times the interquartile range (IQR).
